# Clinical Profile and Predictors of Asthma-Chronic Obstructive Pulmonary Disease Overlap in a Tertiary Care Population in North India

**DOI:** 10.7759/cureus.107788

**Published:** 2026-04-27

**Authors:** Greeshma G R, Varinder Saini, Deepak Aggarwal

**Affiliations:** 1 Department of Respiratory Medicine, Adesh Medical College and Hospital, Shahabad, IND; 2 Department of Pulmonary, Critical Care, and Sleep Medicine, Government Medical College and Hospital, Chandigarh, IND

**Keywords:** absolute eosinophil count, aco: asthma-copd overlap, asthma, copd: chronic obstructive pulmonary disease, spirometry

## Abstract

Background: Asthma and chronic obstructive pulmonary disease (COPD) are common chronic airway disorders with distinct pathophysiology. A subset of patients exhibits overlapping clinical, functional, and inflammatory features, termed asthma-COPD overlap (ACO), which is associated with increased disease burden and management challenges. Data on the prevalence and characteristics of ACO using standardized criteria in the Indian population remain limited. The present study was conducted to determine the prevalence of ACO and to compare its demographic, inflammatory, and functional characteristics with asthma and COPD, and identify factors associated with ACO.

Methods: This cross-sectional study included 200 clinically stable adults with physician-diagnosed asthma or COPD at a tertiary care center in North India. Patients were classified as asthma, ACO, or COPD using the combined Global Initiative for Asthma (GINA) and Global Initiative for Chronic Obstructive Lung Disease (GOLD) clinical and spirometric criteria. Demographic data, smoking exposure, body mass index (BMI), absolute eosinophil count (AEC), spirometry, resting oxygen saturation (SpO_2_), and six-minute walk distance (6MWD) were recorded. Groups were compared, and multivariate logistic regression was used to identify factors independently associated with ACO.

Results: Fifty-one patients (25.5%) met the ACO criteria. The ACO group showed intermediate age, male predominance, and greater smoking exposure. AEC was significantly higher in ACO than in COPD. Post-bronchodilator forced expiratory volume in one second (FEV₁%), SpO_2,_ and 6MWD were lower than in asthma but higher than in COPD. Increasing age, smoking exposure, and higher blood eosinophil count independently predicted ACO.

Conclusion: ACO constituted a considerable proportion of patients among those diagnosed with COPD or asthma. Smoking exposure, persistent eosinophilic inflammation, and functional impairment were intermediate between asthma and COPD.

## Introduction

Asthma and chronic obstructive pulmonary disease (COPD) are among the most prevalent chronic airway diseases in adults and contribute significantly to morbidity, mortality, and healthcare utilization across the world [1,2]. Asthma is characterized by chronic airway inflammation with variable airflow limitation and affects approximately 1-18% of the global population; in India, the prevalence among adults has been reported as 2.05% in the Indian Study on Epidemiology of Asthma, Respiratory Symptoms, and Chronic Bronchitis in Adults (INSEARCH) study [1,3]. COPD is a common, largely preventable disease characterized by persistent airflow limitation and is a leading cause of death worldwide [2]. The disease burden of COPD in India is substantial and represents a significant public health concern.

A proportion of patients with chronic airway disease exhibit a combination of clinical, functional, and inflammatory characteristics of both asthma and COPD. This overlap phenotype was systematically described by Gibson and Simpson, who highlighted its distinct clinical characteristics and outcomes [4]. To describe this entity, the Global Initiative for Asthma (GINA) and Global Initiative for Chronic Obstructive Lung Disease (GOLD) introduced the term “asthma-COPD overlap syndrome (ACOS)” in 2014. The terminology was later modified to “asthma-COPD overlap (ACO)” and defined as the presence of persistent airflow limitation with a combination of features typically associated with asthma and COPD [1,2].

Previous studies have shown that patients with ACO have a higher symptom burden, more frequent exacerbations, poorer quality of life, faster decline in lung function, and increased mortality compared with those having asthma or COPD alone [5-9]. Increased mortality has also been reported in patients with ACO [10]. A structured review of the literature reported that the frequency of ACO among individuals with obstructive airway disease varies widely, ranging from 12% to 55% [5]. Findings from population-based studies across different geographic regions have further highlighted the high prevalence and adverse clinical outcomes associated with this overlap phenotype [11-15].

There is limited data on the prevalence and features of ACO among Indian patients. A study from North India reported that 21.8% of patients previously diagnosed with COPD fulfilled the criteria for ACO [16]. Another study from South India documented an even higher proportion (45%) and observed a greater rate of emergency visits and exacerbations among patients with overlap features [17]. Accurate diagnosis of ACO is important from a treatment perspective as these patients, unlike COPD patients, have high blood eosinophil levels and require inhaled corticosteroids [18,19].

There is a notable lack of prospective Indian studies evaluating ACO among both asthma and COPD populations using the standardized GINA-GOLD diagnostic criteria. Considering the heterogeneity in clinical presentation of ACO across different populations, regional data are essential to better define the clinical profile of ACO [20]. Hence, the present study was conducted to determine the prevalence of ACO among stable, physician-diagnosed asthma and COPD patients attending a tertiary care center and to compare their demographic, clinical, inflammatory, and functional characteristics with those of patients having asthma or COPD alone. 

## Materials and methods

This cross-sectional observational study was conducted at the Department of Pulmonary Medicine, Government Medical College and Hospital, Chandigarh, India, between July 2017 and October 2018. Consecutive adult patients (≥18 years) with a physician diagnosis of bronchial asthma or COPD attending the pulmonary outpatient department and on regular follow-up were screened. Asthma was diagnosed in accordance with the GINA recommendations, using a compatible clinical history along with the demonstration of significant bronchodilator reversibility, defined as an increase in forced expiratory volume in one second (FEV₁) of ≥12% and ≥200 ml after inhaled bronchodilator [1]. COPD was diagnosed using GOLD criteria, based on the presence of persistent airflow limitation with a postbronchodilator FEV₁/forced vital capacity (FVC) ratio <0.70 in a clinically appropriate setting [2]. Regular follow-up was defined as at least two documented outpatient visits in the preceding 12 months with available clinical records and spirometry.

Patients with a history of recent acute exacerbation or lower respiratory tract infection in the last six weeks, concomitant cardiac failure, unstable coronary heart disease, interstitial lung disease, or lung cancer were excluded from the study. Based on previously reported ACO prevalence of approximately 20-30%, a sample size of 200 participants, comprising 100 patients with asthma and 100 patients with COPD, was planned to allow reasonable estimation and group comparisons; however, a formal sample size calculation was not performed [16,17]. Informed written consent was taken from all patients, and the study was approved by the Government Medical College and Hospital, affiliated to Panjab University.

A detailed history was taken from all the patients, specifically describing the type and duration of symptoms, smoking history, occupation, and history of exacerbations. All patients underwent spirometry on a calibrated spirometer (RMS Helios 401; Recorders & Medicare Systems Pvt. Ltd. (RMS), India). Spirometry was performed by trained personnel following standard American Thoracic Society/European Respiratory Society (ATS/ERS) guidelines, with regular calibration of the spirometer to ensure accuracy and reproducibility. Functional exercise capacity was evaluated using the six-minute walk test (6MWT) performed along a 30-meter indoor corridor according to the standard guidelines. Resting SpO₂ was measured by pulse oximetry, and arterial blood gas analysis was performed in patients with SpO₂ of <92%. Hemoglobin concentration and absolute eosinophil count (AEC) were measured using automated hematological analyzers.

Evaluation for ACO was performed using the combined GINA-GOLD guidelines. Clinical features suggestive of asthma (early age of onset, variable symptoms, nocturnal or early morning worsening, atopy, significant bronchodilator reversibility, normal chest radiograph) and those suggestive of COPD (later onset, persistent symptoms, smoking or biomass exposure, chronic cough and sputum, fixed airflow limitation, hyperinflation on chest radiograph) were systematically assessed. Patients were classified using a syndromic approach based on the combined GINA-GOLD framework [1,2]. Clinical features suggestive of asthma and COPD were systematically assessed, and patients were categorized based on the predominant pattern of features. In cases where a comparable number of features of both asthma and COPD were present, a diagnosis of ACO was assigned. This approach, while clinically pragmatic, may introduce some degree of subjectivity. Clinical features, spirometry, six-minute walk distance (6MWD), and arterial oxygen saturation were compared among ACO, asthma, and COPD patients using appropriate statistical tests. Only patients with complete data for the variables included in the analysis were considered, and no imputation for missing data was performed.

Continuous variables were expressed as mean ± standard deviation, and categorical variables as frequencies and percentages. Normality of distribution of continuous variables was assessed using the Shapiro-Wilk test. Comparisons among asthma, ACO, and COPD groups were performed using one-way analysis of variance (ANOVA) for normally distributed variables. Post-hoc pairwise comparisons following ANOVA were performed using independent samples t-tests with Bonferroni correction for multiple comparisons. For categorical variables, pairwise chi-square tests with Bonferroni correction were applied. Multivariate logistic regression analysis was performed to identify independent predictors of ACO. Variables entered into the model included age, sex, body mass index (BMI), smoking pack-years, post-bronchodilator FEV₁ % predicted, and AEC. A p-value <0.05 was considered statistically significant. Multicollinearity among independent variables was assessed using the variance inflation factor (VIF), with a value of <5 considered acceptable. All analyses were conducted using IBM SPSS Statistics for Windows, Version 21 (Released 2012; IBM Corp., Armonk, New York, United States).

## Results

A total of 200 clinically stable adults with obstructive airway disease were included in the final analysis. Based on the combined GINA-GOLD clinical and spirometric criteria, 66 patients were classified as asthma, 51 as ACO, and 83 as COPD. Thus, ACO constituted 25.5% of the study population.

Patients with ACO were significantly older than those with asthma and slightly younger than those with COPD (57.2 ± 9.9 vs 38.8 ± 13.7 and 60.9 ± 7.6 years, respectively; p < 0.001). The proportion of males was significantly higher in the ACO and COPD groups compared to the asthma group (p < 0.001). Smoking exposure showed a graded increase across the three groups, being lowest in asthma (1.5 ± 5.4 pack-years), intermediate in ACO (20.9 ± 21.9 pack-years), and highest in COPD (47.9 ± 32.1 pack-years) (p < 0.001) (Table 1).

**Table 1:** Baseline demographic and clinical characteristics (N = 200) ACO: asthma-COPD overlap; BMI: body mass index; COPD: chronic obstructive pulmonary disease; ANOVA: analysis of variance Continuous variables were compared using one-way ANOVA, and categorical variables using the chi-square test

Variable	Asthma (n = 66)	ACO (n = 51)	COPD (n = 83)	Test statistic	p-value
Age (years)	38.8 ± 13.7	57.2 ± 9.9	60.9 ± 7.6	F = 89.82	<0.001
Male sex, n (%)	34 (51.5)	31 (60.8)	69 (83.1)	X^2 ^= 25.24	<0.001
Pack-years	1.5 ± 5.4	20.9 ± 21.9	47.9 ± 32.1	F = 75.63	<0.001
BMI (kg/m²)	22.8 ± 4.4	21.7 ± 3.8	21.4 ± 4.0	F = 3.27	0.10
Hemoglobin (g/dL)	12.8 ± 2.0	13.1 ± 1.8	13.5 ± 1.7	F = 3.38	0.18

Post-hoc analysis using Bonferroni correction demonstrated that age was significantly lower in asthma compared to both ACO and COPD (p < 0.001), while no significant difference was observed between ACO and COPD. Smoking exposure (pack-years) differed significantly across all groups, showing a graded increase from asthma to ACO to COPD (p < 0.001 for all pairwise comparisons). Male sex distribution did not differ significantly between asthma and ACO (p = 0.517), but was significantly higher in COPD compared with asthma (p < 0.001) and ACO (p = 0.010) (Table 2).

**Table 2:** Post-hoc pairwise comparisons of baseline variables ACO: asthma-COPD overlap; COPD: chronic obstructive pulmonary disease; ANOVA: analysis of variance Pairwise comparisons for age and pack-years were performed using independent samples t-tests with Bonferroni correction for multiple comparisons following one-way ANOVA. Pairwise comparisons for male sex were performed using chi-square tests with Bonferroni correction. All p-values represent Bonferroni-adjusted values

Comparison	Age (years) p-value	Pack-years p-value	Male sex p-value
Asthma vs ACO	<0.001	<0.001	0.517
Asthma vs COPD	<0.001	<0.001	<0.001
ACO vs COPD	0.18	<0.001	0.010

AECs differed significantly among the three groups (p < 0.001). Patients with asthma had the highest mean eosinophil levels (640 ± 575 cells/µL), followed by those with ACO (523 ± 433 cells/µL), while COPD patients showed markedly lower values (174 ± 137 cells/µL). The inflammatory profile of ACO therefore remained closer to asthma than to COPD, suggesting persistence of eosinophilic airway inflammation in a substantial proportion of overlap patients. Post-bronchodilator FEV₁ (% predicted) demonstrated a stepwise decline from asthma to ACO and COPD. Mean values were highest in asthma (91.5 ± 24.7%), intermediate in ACO (64.4 ± 20.5%), and lowest in COPD (49.9 ± 16.9%) (p < 0.001), indicating persistent airflow limitation with partial reversibility in the overlap group. Patients with asthma walked the longest distance (398 ± 58 m), followed by those with ACO (344 ± 63 m), while the shortest distances were observed in COPD (323 ± 68 m). Patients with asthma demonstrated the best functional capacity, followed by ACO and COPD, indicating a graded decline in exercise performance across groups (Table 3).

**Table 3:** Comparison of functional and inflammatory parameters (N = 200) AEC: absolute eosinophil count; ACO: asthma-COPD overlap; COPD: chronic obstructive pulmonary disease; FEV₁: forced expiratory volume in one second; post-BD: post-bronchodilator; SpO₂: peripheral oxygen saturation; 6MWD: six-minute walk distance; ANOVA: analysis of variance Continuous variables were analyzed using one-way ANOVA with Bonferroni post-hoc correction

Parameter	Asthma (n = 66)	ACO (n = 51)	COPD (n = 83)	Test statistic	p-value
AEC (cells/µL)	640 ± 575	523 ± 433	174 ± 137	F = 23.93	<0.001
SpO₂ (%)	96.4 ± 2.8	94.9 ± 3.0	94.0 ± 4.4	F = 5.08	<0.01
6MWD (m)	398 ± 58	344 ± 63	323 ± 68	F = 24.35	<0.001
Post-BD FEV₁ %	91.5 ± 24.7	64.4 ± 20.5	49.9 ± 16.9	F = 81.81	<0.001

Post-hoc analysis revealed that the AEC was significantly higher in asthma and ACO compared to COPD (p < 0.001), while no significant difference was observed between asthma and ACO. Functional parameters, including 6MWD and post-bronchodilator FEV₁, showed significant differences across all pairwise comparisons, with a graded decline from asthma to ACO to COPD (Table 4).

**Table 4:** Post-hoc pairwise comparisons of inflammatory and functional parameters AEC: absolute eosinophil count; 6MWD: six-minute walk distance; FEV₁: forced expiratory volume in one second; post-BD: post-bronchodilator; ACO:  asthma-COPD overlap; COPD: chronic obstructive pulmonary disease; ANOVA: analysis of variance Pairwise comparisons for AEC, 6MWD, and post-bronchodilator FEV₁ were performed using independent samples t-tests with Bonferroni correction for multiple comparisons following one-way ANOVA. All p-values represent Bonferroni-adjusted values

Comparison	AEC (cells/µL) p-value	6MWD (m) p-value	Post-BD FEV₁ (%) p-value
Asthma vs ACO	0.32	<0.001	<0.001
Asthma vs COPD	<0.001	<0.001	<0.001
ACO vs COPD	<0.001	0.03	<0.001

On multivariate logistic regression analysis, increasing age was independently associated with the presence of ACO (adjusted OR: 1.07 per year; 95% CI: 1.03-1.10; p < 0.001). Smoking exposure also showed a significant association when expressed per 10 pack-year increment (adjusted OR: 1.18; 95% CI: 1.03-1.35; p = 0.016). Higher AEC remained an independent predictor (adjusted OR: 1.10 per 100 cells/µL; 95% CI: 1.02-1.18; p = 0.004). Gender and body mass index were not independently related to ACO after adjustment (Table 5).

**Table 5:** Multivariate logistic regression analysis for predictors of ACO AEC: absolute eosinophil count; BMI: body mass index; CI: confidence interval; OR: odds ratio; COPD: chronic obstructive pulmonary disease; ACO: asthma-COPD overlap AEC expressed per 100 cells/microliter increment. Reference category: Non-ACO group (asthma and COPD). For male sex, the reference category was female

Variable	Adjusted OR	95% CI	p-value
Age (per year)	1.07	1.03-1.10	<0.001
Pack-years (per 10)	1.18	1.03-1.35	0.016
AEC (per 100 cells/µL increase)	1.10	1.02-1.18	0.004
BMI (Kg/m^2^)	0.93	0.85-1.02	0.12
Male sex	1.62	0.66-3.98	0.29

The regression model demonstrated good calibration (Hosmer-Lemeshow p > 0.05) and acceptable explanatory power (Nagelkerke R² ≈ 0.41), with no evidence of significant multicollinearity among covariates (all VIF values < 2). Collectively, these findings indicate that older age, cumulative smoking exposure, and persistence of eosinophilic inflammation are key independent determinants of the ACO phenotype, which functionally and physiologically lies between asthma and COPD.

## Discussion

In this tertiary care cohort, about one-quarter of adults with obstructive airway disease met criteria for ACO, indicating that the overlap phenotype forms a significant proportion of patients seen in routine clinical practice. This frequency is comparable to the wide prevalence range reported in earlier systematic reviews and population-based studies, where ACO has been observed in 12-55% of patients with chronic airflow limitation, highlighting its clinical relevance [5,11].

The demographic characteristics of patients with ACO in the present study lie between those of asthma and COPD, with respect to age and smoking exposure, a pattern that has also been reported in international cohorts. Similar findings of older age and significant smoking history among overlap patients have been reported in previous studies [16,18]. Long-term smoking may lead to structural airway changes that contribute to the development of fixed airflow limitation in individuals with underlying asthma. Large population-based studies from Europe, North America, and Asia have also demonstrated that patients with overlap exhibit characteristics of both diseases, reflecting the heterogeneity of this phenotype [11-15].

Functional impairment in ACO, assessed by 6MWD, was intermediate between asthma and COPD in our cohort, even after adjustment for age. Previous studies have reported that patients with overlap have poorer exercise tolerance and quality of life than those with asthma alone, though less severe limitation than in advanced COPD [11]. This reduction in exercise capacity may be due to a combination of fixed airflow obstruction, dynamic hyperinflation, and associated comorbidities, as suggested by previous physiological studies [21].

Inflammatory profiling revealed that blood eosinophil counts in ACO were significantly higher than in COPD and closer to those in asthma, indicating the persistence of type 2 airway inflammation in a substantial subset of overlap patients. Several studies have reported that eosinophilic inflammation is common in ACO and may have therapeutic implications [19,22]. Higher blood eosinophil levels are associated with an increased risk of exacerbations and a better response to inhaled corticosteroid therapy in asthma and eosinophilic COPD, supporting their role as a clinically useful, though imperfect, biomarker in the overlap spectrum [22].

On multivariate analysis, increasing age, cumulative smoking exposure, and higher blood eosinophil counts showed independent association with ACO. These findings are consistent with earlier Indian studies, which identified smoking and markers of eosinophilic inflammation as key determinants of overlap in Indian populations [16,17]. 

Beyond cross-sectional characterization, several studies have shown that ACO is associated with higher symptom burden, more frequent exacerbations, increased healthcare utilization, and impaired quality of life compared with asthma or COPD alone. Multiple studies have reported lower quality of life, higher hospitalization rates, and increased risk of exacerbations among patients with overlap compared with asthma or COPD alone [6-9]. A recent clinical review also emphasized the heterogeneity of ACO phenotypes and the need for individualized management strategies in routine practice [23]. More recent studies and cohort analyses have further highlighted the heterogeneity and clinical complexity of this phenotype [24].

Accurate recognition of ACO also has important implications from a therapeutic point of view. Previous authors have highlighted the importance of inhaled corticosteroids in patients with asthma features or eosinophilic inflammation and cautioned against the use of long-acting bronchodilator monotherapy in this group [18]. Emerging evidence suggests that biologic agents targeting type two inflammation, such as dupilumab, may reduce exacerbations and improve outcomes in COPD patients with elevated eosinophil counts, although data specific to well-defined ACO populations remain limited [25].

The present study provides insight into the high prevalence of ACO in patients being treated for asthma or COPD. The distinct clinical features, objective parameters (6MWD and FEV1), and inflammatory biomarkers of ACO highlight the need for specific treatment requirements different from those of asthma and COPD patients. However, the study has certain limitations as well. First, the cross-sectional nature of the analysis precludes assessment of longitudinal outcomes such as treatment response, exacerbation frequency, rate of lung function decline, and mortality. Secondly, advanced biomarkers of airway inflammation, including fractional exhaled nitric oxide, sputum cytology, and immunoglobulin E levels, were not evaluated, which might have allowed further phenotypic and endotypic characterization. The potential for classification bias and limited external validity due to the single-center tertiary care setting should be considered when interpreting these findings. Furthermore, the lack of a universally standardized definition of ACO and reliance on a syndromic classification approach may introduce subjectivity and limit reproducibility. Additionally, the absence of a formal sample size calculation and the use of complete-case analysis without imputation for missing data may have introduced bias. Finally, treatment response and long-term therapeutic outcomes in patients with ACO were not assessed and warrant evaluation in future prospective studies.

## Conclusions

The present study confirms that ACO is a common and clinically important phenotype among patients with obstructive airway disease in the Indian setting. Patients with overlap demonstrate demographic, inflammatory, and functional characteristics that are distinct yet intermediate between asthma and COPD. Older age, smoking exposure, and persistence of eosinophilic inflammation appear to be key determinants of this phenotype. These findings should be interpreted in the context of the cross-sectional design, and the identified predictors represent associations rather than causal relationships.
